# Embodied Energy Optimization of Prestressed Concrete Road Flyovers by a Two-Phase Kriging Surrogate Model

**DOI:** 10.3390/ma16206767

**Published:** 2023-10-19

**Authors:** Lorena Yepes-Bellver, Alejandro Brun-Izquierdo, Julián Alcalá, Víctor Yepes

**Affiliations:** 1 Mechanics of Continuous Media and Theory of Structures Department, Universitat Politècnica de València, 46022 Valencia, Spain; loyebel@alumni.upv.es; 2 School of Civil Engineering, Universitat Politècnica de València, 46022 Valencia, Spain; albruiz1994@gmail.com; 3 Institute of Concrete Science and Technology (ICITECH), Universitat Politècnica de València, 46022 Valencia, Spain; jualgon@cst.upv.es

**Keywords:** optimization, embodied energy, bridges, surrogate model, Kriging, prestressed concrete, sustainability

## Abstract

This study aims to establish a methodology for optimizing embodied energy while constructing lightened road flyovers. A cross-sectional analysis is conducted to determine design parameters through an exhaustive literature review. Based on this analysis, key design variables that can enhance the energy efficiency of the slab are identified. The methodology is divided into two phases: a statistical technique known as Latin Hypercube Sampling is initially employed to sample deck variables and create a response surface; subsequently, the response surface is fine-tuned through a Kriging-based optimization model. Consequently, a methodology has been developed that reduces the energy cost of constructing lightened slab bridge decks. Recommendations to improve energy efficiency include employing high slenderness ratios (approximately 1/28), minimizing concrete and active reinforcement usage, and increasing the amount of passive reinforcement.

## 1. Introduction

The construction industry is a pivotal driver of economic growth in numerous countries worldwide. Nevertheless, this substantial role in the global economy places the construction sector at the forefront of concerning aspects, including non-renewable resource consumption, waste generation, and the production of greenhouse gas (GHG) emissions. Construction constitutes a significant portion of global energy consumption, ranging from 25% to 40% [1]. Assessing sustainability in construction is often based on the energy expended during construction and the resulting CO_2_ emissions [2,3]. Consequently, there has been a growing focus on improving environmental sustainability within the construction sector in recent years [4].

Furthermore, studies showed that the cement production industry accounts for approximately 5% of all energy consumption within the industrial sector [5]. The challenge is balancing descriptive regulations and performance-based approaches in cement and concrete standardization. This combination would facilitate the adoption of new technical solutions while guaranteeing both durability and sustainability [6]. A pivotal move toward achieving environmental sustainability involves transitioning from cement-type CEM I to CEM II (European classification) [7]. Cabeza et al. [8] provided an overview of embodied carbon and energy values by conducting a keyword analysis and systematically reviewing literature data. This analysis unveiled concrete as the most assessed material and the use of the “cradle to gate” boundary system in Life Cycle Assessments (LCA). In the context of LCAs, it is assumed that the structure has undergone wear and tear during its operation and will necessitate repair and maintenance, typically involving conventional materials and methods [9,10]. It is crucial not only to conduct an analysis that solely focuses on economic or environmental aspects throughout a life cycle but also, as emphasized by Navarro et al. [11], to comprehensively assess social sustainability across an entire structure’s life cycle. However, the literature indicates the significance of each impact also considerably varies depending on the type of structure [12].

Proper material selection and optimization contribute to the sustainability of concrete structures [13]. Meng et al. [14] developed an optimization design for ultra-high-performance concrete. Meanwhile, Kim et al. [15] assessed CO_2_ emissions throughout the concrete’s life cycle and introduced an optimal design method based on an evolutionary algorithm. Structural engineering has traditionally focused on cost-effective safety solutions that minimize investments. However, the current emphasis on sustainable development demands a departure from this criterion as it proves inadequate in aligning with broader sustainability objectives. In response, alternative criteria have emerged to integrate sustainability principles into structural design [16]. Some authors have made energy a central focus in their optimization endeavors [17,18]. Miller et al. [19] demonstrated that in situ construction methods for various slab systems exhibit lower energy consumption compared to reinforced concrete slabs. Energy reduction results in reduced structural weight, but weight reduction does not necessarily lead to the lowest achievable energy consumption [20]. However, research dedicated to embodied energy optimization in bridges is scarce. Minunno et al. [21] provide a regression model and procedural guidelines for practitioners seeking to reduce buildings’ environmental impact. This study suggests adopting modularized and disassemblable building construction systems. Penadés-Plà et al. [22] introduced an optimization algorithm for a three-span footbridge with lengths of 40-50-40 m.

Heuristic optimization has proven to be a valuable tool in mitigating the economic and environmental costs associated with structural engineering [23,24,25,26,27]. Nevertheless, these techniques can be computationally expensive, leading to the use of metamodels to tackle this challenge. To avoid the limitations of previous studies based on heuristic optimization, this paper employs a surrogate model instead of an intricate one for optimization simulations. This approach can be employed to streamline the optimization process, explore the design space, or conduct reliability analysis, among other applications. The core methodology entails obtaining a sample set of design vectors within the design space, followed by the execution of high-fidelity simulations, such as Finite Element Analysis (FEA). Subsequently, regression or interpolation models are constructed based on the high-fidelity values, and these models can be scrutinized using optimization algorithms [28]. Employing this approach with effective optimization techniques offers the advantage of swiftly achieving optimal results. The metamodels most frequently employed comprise polynomial regression, Radial Basis Functions (RBFs), Neural Networks (NN), and Kriging models [29,30].

One of the most effective metamodels is Kriging, which replaces a simulation model and provides optimal interpolation using observed values [31]. Kriging-based optimization is presented as a distinct approach from heuristic optimization to accelerate complex problems’ optimization [22]. The Kriging surrogate model is a nonparametric interpolation model that posits the actual performance function as a manifestation of a Gaussian process [32]. This model utilizes the training samples and their associated outputs to build the surrogate model, enabling predictions for unobserved points. The Kriging methodologies construct a metamodel by employing optimal interpolation techniques through regression against observed values from adjacent data points, with weights determined by spatial covariance values [22].

Nevertheless, only a few studies have applied Kriging to real structural design problems. Martínez-Frutos and Martí [33] propose an approach using Kriging surrogate models to solve in a very efficient manner the uncertainty assessment problem in optimizing the design of robust structures. Recently, it has been utilized in optimizing wind turbines [34], auxiliary structures for circular bridge piers [35], and reinforced concrete frame structures [36,37].

Zhang and Wu [38] utilized Kriging in RC bridges for establishing the structural vulnerability curves. A Kriging-based algorithm reduced computing time by 99.06% while yielding results deviating only 2.54% from the approach using heuristic optimization with simulated annealing [22]. Wu et al. [39] applied this model to optimize bridge structure finite element models.

This article presents a general methodology for reducing energy consumption in constructing lightened prestressed concrete (PC) slab bridges, particularly in post-tensioned road flyovers. This approach can be applied to other structures for optimizing various objective functions. The benefit of adopting this approach lies in its capacity to address intricate problems characterized by many variables, especially in cases where computational constraints lead to extended processing times. The proposed method is universally applicable and can be used in several structural contexts to optimize objective functions. Two novel contributions are highlighted: adopting a two-phase Kriging metamodel and optimizing the embodied energy in multiple voided PC slab decks.

## 2. Problem Description

Employing a continuous hyperstatic PC slab in bridges between 10 and 45 m is common practice. Slab decks are no longer cost-effective beyond a main span of 50 m, resulting in a transition to box girder cross-section decks in the design. In common practice, when designing slab decks for roads with three or more span segments, the depth-to-span ratio is typically maintained at approximately 1/25. This solution competes favorably with prefabricated beams due to its structural advantages, including increased torsional and flexural rigidity, enhanced durability, and safety attributed to hyperstatic behavior. Additionally, it easily adapts to complex shapes from a construction perspective, simplifying the formwork and concrete pouring processes. Furthermore, it eliminates joints and provides greater flexibility in support placement, all while enhancing aesthetics.

**Figure 1:**
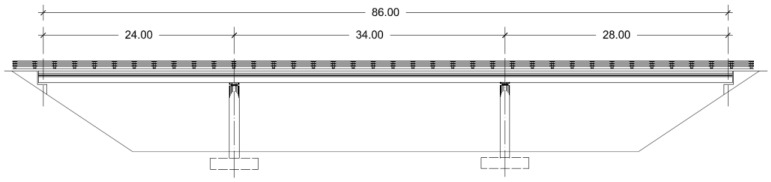
Longitudinal profile of the PC slab road bridge.

**Figure 2:**
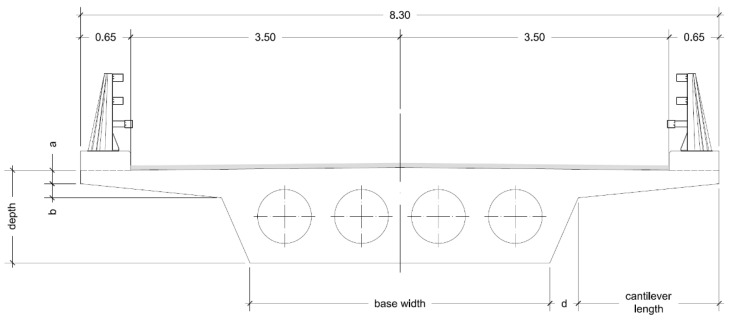
Cross-sectional view of the lightweight PC slab bridge deck.

The objective of this study is to optimize the embodied energy of prestressed concrete road flyovers using a two-phase Kriging surrogate model. To achieve this, this new metamodel has been applied to improve the prestressed lightweight slab bridge deck design with spans measuring 24-34-28 m, a configuration commonly found in flyovers spanning double-lane and double-track motorways. As depicted in Figure 1, the cast-in-place slab maintains a uniform depth and follows a straight-line layout. The deck has a width of 8.30 m, accommodating two 3.50 m lanes, a 0.65 m guard rail on each side, and a concrete pedestal (see Figure 2).

The theory of limit states is employed to assess structural strength using partial safety factors. Each design scenario ensures that no limit state, whether ultimate or related to serviceability, is surpassed. In this instance, CSiBridge v.21.0.0 software was employed to model, analyze, and size the bridge deck. Each alternative was analyzed to determine the acting and resisting forces represented by sectional stresses. The structures are verified according to the serviceability limit state (SLS) and the ultimate limit state (ULS) defined in Eurocode 2, considering the actions specified in Eurocode 1, which include dead loads of 44 kN/m and the environmental exposure class of concrete XC4. These demands are derived for each structural element and are detailed in [40].

The variables under consideration included the concrete’s simple compressive strength and depth and width dimensions on the various decks’ cross-sections (Figure 2). The remaining dimensions and lightweight components are derived from the relationships outlined in Table 1. Consequently, the concrete’s characteristic strength was modified, ranging from 30 to 50 MPa. The depth ranges from 1.15 m to 1.70 m, with 0.05 m increments, while the width of the section ranges from 3.00 m to 5.00 m, with 0.05 m increments. The maximum span length of the cantilever (*v*) depends on the bottom base (*b*), creating an interdependence between these variables. This interrelation can be problematic, as extended cantilever lengths may not be compatible with specific bottom width values, where the combined summation of the bottom width and the lengths of the cantilever should be, at most, the total width of the deck. The voids under consideration are consistently circular, and the external cross-section solely determines their placement within the cross-section. The shear reinforcement and the prestressing tendons need to be positioned within the void cross-section webs. Achieving the required prestressing level may necessitate employing more than one cable per web. It is necessary to integrate the shear reinforcement with the prestressing layout.

## 3. Methodology

The proposed methodology comprised two phases: diversification and intensification. Latin Hypercube Sampling (LHS) was employed to select uniformly distributed random numbers. This method was introduced by McKay et al. [41], demonstrating that Latin Hypercube Sampling (LHS) yields a lower variance of the sample mean compared to a simple random sample. The embodied energy for each alternative is evaluated and then optimized using a response surface created by a Kriging metamodel. LHS randomly selected a sample within each interval for every variable, and the numerical model is executed as often as there are intervals in the probability distribution division. This ensured the selection of initial values within each data range.

LHS stands out for several key advantages. It offers a more comprehensive understanding of the design space than simple random sampling. It is especially advantageous for computational experiments focusing on systematic rather than random errors and ensures a uniformly random sample. In addition, LHS provides the flexibility to adjust the sample size to suit specific experimental needs. Furthermore, it excels in ease of generation and delivers efficient results in a reasonable time frame, making it a practical choice for various applications.

The sampling process depends on the size and placement of selected points. The sample size proportionally increases with the number of variables to maintain the same metamodel precision. For concrete structures, favorable results have been obtained with a sample size of 30 individuals. Subsequently, the values were fitted within the ranges of each variable through this sampling process, generating designs that serve as inputs in the optimization model.

Each bridge deck consumes energy, and to compare different designs, various elements were analyzed, including concrete type, formwork area, steel quantity, and lightweight volume (Table 2).

While a typical study would generally choose the deck with the lowest energy requirement, a predictive Kriging-type model was proposed to optimize the alternatives resulting from sampling using a heuristic algorithm. Kriging operates by interpolating data through regression analysis using observed values from nearby points, with weights determined by spatial covariance. The model simultaneously considers global and local approximations, allowing for a consideration of local variations in the response.

Kriging is based on predicting the attribute value, represented as *z*, at a point *u*, using *n* values of *z*. In this case, the attribute represents the energy required for deck construction, while the points correspond to the points obtained through LHS sampling. This process predicts the response without the need for a complete structural analysis. The MATLAB Kriging Toolbox (DACE), which constructs a Kriging model from data generated in a computer experiment, was employed, consisting of pairs of inputs and model responses [42].

**Figure 3:**
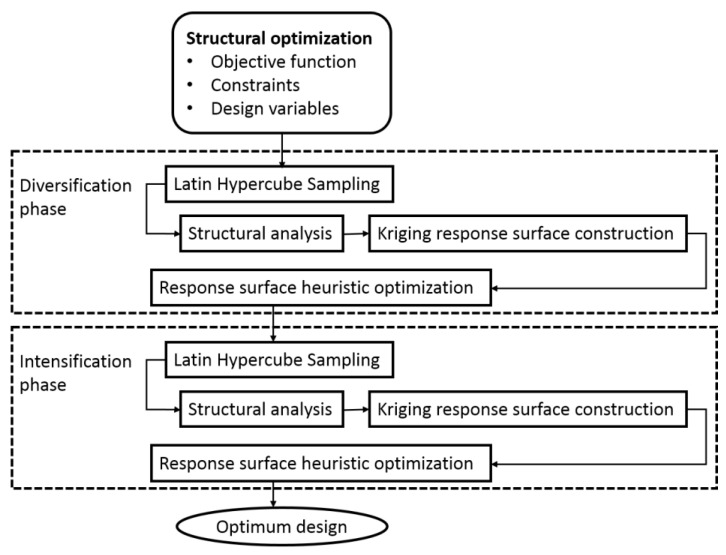
Metamodel-based heuristic optimization flowchart.

To achieve higher accuracy in the response, the slowest part of conventional optimization, namely structural analysis and objective function evaluation, is substituted with metamodel predictions. As a result, the computational cost for metamodel-based optimization is significantly reduced compared to conventional methods. For example, Penadés-Plà et al. [22] found that Kriging can reduce computational cost by 99.06%. This model’s use allows for the resolution of computationally intensive structural problems while simplifying the complexity of other issues. Figure 3 illustrates the process flow diagram.

Threshold Accepting with Mutation Operator (TAMO) [43] was employed in this study. This method is a simulated annealing (SA) derivative and can be classified as a local search technique for tackling non-deterministic polynomial-time hard (NP-hard) optimization problems spanning various domains. Much like SA, which employs a probabilistic criterion to escape local optima, TAMO is a search method that involves making slight random modifications to the current solution and systematically navigating the search space. This metaheuristic permits deteriorating moves to break free from local optima. This is achieved by accepting solutions that degrade the current solution by a specified threshold, progressively reducing this threshold to zero. This allows for accepting modifications that may lead to worse outcomes to avoid local minima. This algorithm is guided by four key rules: the initial solution must be generated at the beginning, a set of neighbors should be defined to generate neighboring solutions, criteria for stopping the search must be chosen, and tuning the threshold value is a crucial aspect of the process. This algorithm begins with an initial random solution and a starting threshold. Following Medina’s criterion [44], the initial threshold (*U*_0_) is adjusted until it falls within the 20% to 40% acceptability range. The new solution can be altered in each iteration, simulating the mutation process seen in genetic algorithms. This modification introduces an exploratory element into the optimization process. The initial threshold is geometrically reduced after every 1000 iterations, with an 80% cooling coefficient. This algorithm is used for optimizing structures due to its strong convergence towards the global optimum [45].

## 4. Results

### 4.1. Search Diversification Phase

Latin Hypercube Sampling (LHS) is employed to explore local optima in a diversified way in the initial phase. After determining the design variables, an LHS sample is extracted to obtain various permutations of these variables, contributing to the surrogate model. The values obtained are between 0 and 1. Nonetheless, it is assumed that the depth and width of the bottom are multiples of 0.05 m. In addition, concrete grade values are restricted to integer multiples of 5. As a result, the ultimate dimensions for the different bridge design solutions under consideration are outlined in Table 3.

The data selected in this sampling process served as input for the Kriging model. The analysis and verification of the bridge decks were examined and verified, considering both ultimate limits and serviceability states while calculating energy consumption.

Relevant elements contributing to energy consumption were evaluated to facilitate a comparison among the various bridge decks. These elements included the concrete grade employed, the required formwork surface area, the volume of lightweight materials, and the quantity of active and passive steel used. The energy consumption was correlated with the measurements of each material, as illustrated in Table 4.

A conventional analysis of solutions would lead to the selection of Deck #3, as it results in the lowest energy cost. However, in pursuit of an even more efficient solution, a Kriging model for optimization was employed. With the assistance of simulated annealing, an optimal response surface has been achieved, and the outcomes are summarized in Table 5.

### 4.2. Search Intensification Phase

This phase aimed to intensify the search process for optimal results based on the better solution achieved in the diversification phase. In this stage, the variables have been narrowed down close to the best solution from the preceding phase, and an additional ten individuals have been added to assess if further improvements can be achieved. After analyzing the new bridge decks, the constraints mandated by regulations were verified, and the energy assessment was conducted as outlined in Table 6.

As is evident, the best bridge deck from the diversification phase showcased a percentage reduction in the values of all sampled decks, ranging from 1.5% in the best case to as much as 17.1% in the worst case.

Introducing new individuals was optional to outperform the best result identified during the diversification phase. Nevertheless, after optimizing the modified response surface, a new optimum surpassed the former (as shown in Table 7). When comparing the energy result obtained after the optimization of this second phase, a reduction of up to 1.21% is achieved.

## 5. Discussion

For prestressed slab bridges with cantilevers, the recommendations from the Direccón General de Carreteras (DGC) [46] suggest a slenderness ratio between 1/22 and 1/30, while SETRA [47] recommends a slenderness ratio of 1/28 for three-span slab decks with wide cantilevers. In our case, the slenderness ratio of the bridge optimized for energy efficiency is 1/29.57, which falls within the limits recommended by DGC [46] and is very close to the limit set by [47].

The deck optimization reduces the depth, resulting in these bridges’ very high slenderness ratio. When comparing the slenderness ratio of the bridge optimized for energy with the data from the study by Yepes et al. [48], which statistically analyzed 61 lightweight slabs, it can be observed that the slenderness ratio exceeds the 75th percentile (1/26.39). Only one deck in that study had a slenderness ratio greater than 1/30, suggesting that the design of such slender decks is uncommon.

Regarding the amount of concrete, DGC [46] suggests a range of 0.55 to 0.70 m^3^/m^2^ for the deck. The energy-optimized deck has a quantity of 0.60 m^3^/m^2^, falling within this recommendation. When comparing this concrete quantity with the data provided by [48], it is slightly below the sample’s median.

**Figure 4:**
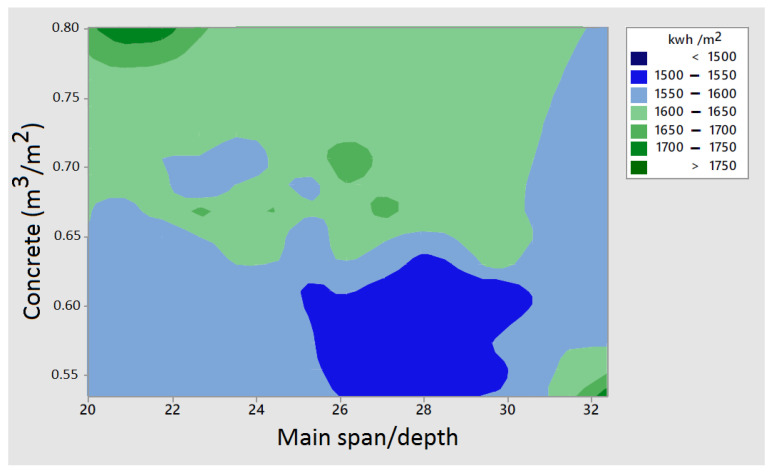
Contour plot of embodied energy with respect to main span/depth and concrete quantity.

Another relevant consideration involves examining the relationship of depth/main-span ratio to the concrete quantity for all the bridges analyzed. As depicted in Figure 4, it becomes evident that higher slenderness ratios, between 1/26 and 1/30, along with minimal concrete volumes, less than 0.60 m^3^/m^2^, hold significance.

The quantity of passive steel employed in the energy-optimized deck surpasses the DGC [46] recommendations by 12%, establishing a range of values between 70 and 100 kg/m^3^ of passive steel to the volume of concrete. The optimized bridge requires 112.32 kg/m^3^ of passive steel, representing a substantial deviation from the recommended values.

However, the median value from [48] is 100.87 kg/m^3^, close to the typical amount of passive steel used in bridges previously constructed in Spain. It can also be noted that the values provided by the DGC [46] fall below what has been executed.

The quantity of passive steel in the bridge optimized for energy consumption is lower than the maximum value found in [48], which was 187.08 kg/m^3^. Furthermore, the amount of passive reinforcement per unit of bridge area is close to the median in the same study. The energy-optimized bridge uses an amount of 67.32 kg/m^2^ of the deck, while the median is 65.27 kg/m^2^ of the deck.

**Figure 5:**
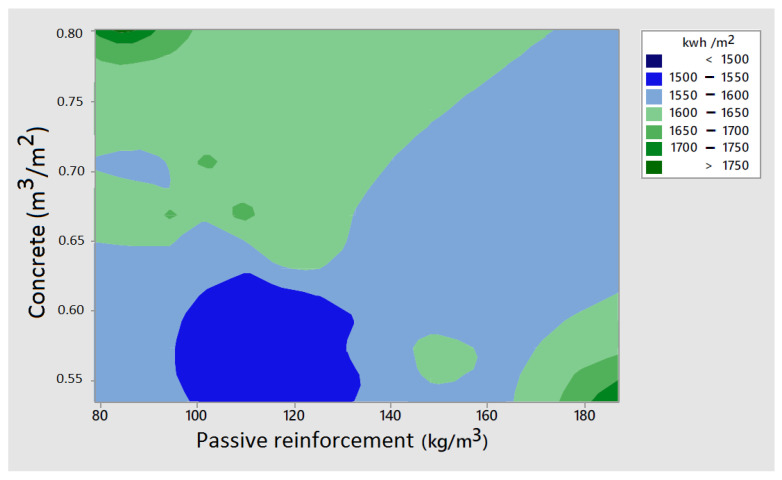
Contour plot of embodied energy with respect to passive reinforcement and concrete quantity.

Figure 5 presents the variation in embodied energy concerning the concrete quantity and the passive reinforcement for all the bridges analyzed. Energy consumption decreases with concrete quantities below 0.60 m^3^/m^2^ and passive reinforcement quantities ranging from 100 to 130 kg/m^3^.

As for the quantity of active reinforcement, the optimized bridge consumes 16.48 kg/m^2^ of deck, which falls within the limits established by the DGC [46] of 10 to 25 kg/m^2^. However, this value is below the 25th percentile of the work by [48], suggesting that energy-optimized slab bridges tend to reduce the amount of prestressing and concrete in exchange for increasing the amount of passive reinforcement.

**Figure 6:**
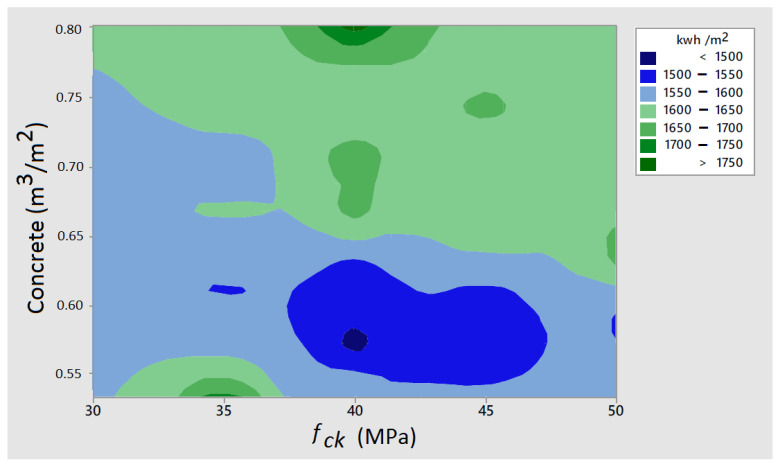
Contour plot of embodied energy with respect to concrete grade and its quantity.

Another aspect worth exploring is the relationship between the characteristic strength of concrete and its quantity. Figure 6 shows lower energy consumption occurs with concrete quantities ranging from 0.55 to 0.60 m^3^/m^2^, and the concrete strength is approximately 40–45 MPa.

## 6. Conclusions

The conclusions drawn from the conducted study are presented below. It is essential to highlight that in this work, a methodology for optimizing structures using metamodels has been developed, allowing for the systematic and efficient resolution of an existing problem. The proposed methodology has been applied to a 24-34-28 m three-span lightweight slab PC bridge deck.

First and foremost, the research question has been addressed by providing a two-phase methodology founded on a Kriging surrogate model, which improves the energy cost associated with constructing a lightweight prestressed slab deck. This methodological proposal allows for its use in optimization problems that involve high computational costs when using heuristic algorithms. This approach is particularly relevant due to the many variables and constraints in real structural problems. On the other hand, optimizing the embedded energy required to construct a structure, such as the studied overpass, highlights the potential for designing more sustainable structures. This approach, therefore, allows us to discern the design differences between solutions that require less embedded energy compared to the typical pre-dimensioning rules used in conventional practice.

From the analysis of the results, the conclusions drawn are as follows:

- Designing slab decks to reduce energy consumption involves a slight increase in the typical slenderness ratios of these elements. This action reduces the volume of concrete used and the amount of prestressing required while increasing the consumption of passive reinforcement. Furthermore, energy consumption can be reduced by decreasing the volume of materials such as steel for active reinforcement and concrete. It is recommended to use wide cantilevers and lightweight interior materials with the maximum height possible within the design to achieve this, allowing for a reduction in the volume of concrete used.
- The following recommendations are made to reduce emissions in a three-span prestressed slab bridge with a main span of 34 m: maintaining a slenderness ratio of approximately 1/28, employing a concrete volume ranging from 0.55 to 0.60 m^3^/m^2^ for the deck, using passive steel in quantities between 100 and 130 kg/m^3^, incorporating active reinforcement at around 17 kg/m^2^ of deck, specifying a characteristic concrete strength of 40 MPa, keeping interior lightweight material quantities below 0.18 m^3^/m^2^ of deck, and using exterior lightweight material quantities ranging from 0.45 and 0.55 m^3^/m^2^ of deck.

The work is constrained by its focus on optimizing a single objective function, specifically embedded energy. Therefore, it would be advantageous to investigate the concurrent integration of additional objective functions, such as cost, CO_2_ emissions, and safety, alongside embedded energy for future research directions. Furthermore, this methodology could be extended to various structural configurations. Another promising avenue for research entails analyzing these objective functions throughout the entire life cycle of the structure, encompassing maintenance and eventual dismantling. Future research holds the potential to enhance this subject by advancing the modeling of in-service longevity. It can achieve this by employing various repair technologies to ascertain which strategy yields the most significant extension in the lifespan of bridges.

## Figures and Tables

**Table 1:** Dimensional ranges and their regulatory constraints [40].

Design Variables	Range	Limitation
Base width (*b*)	3.00–5.00 m	-
Depth of the deck (*c*)	1.15–1.70 m	>0.90 m
Length of the cantilever (*v*)	Variable	<3.50 m
Initial cantilever thickness *e*_1_ (*a* + *b*)	0.35 m	-
Thickness of the cantilever edge *e*_2_ (*a*)	0.25 m	>0.20 m
Distance between cantilever and core (*d*)	0.40 m	-
Minimum void coating	0.225 m	>0.15 m
Characteristic strength of concrete (*f_ck_*)	30–50 MPa	-

**Table 2:** Energy cost of the deck [22].

Material	kWh/kg	kWh/m^3^	kWh/m^2^
Y-1860-S7 steel	5.64		
B-500-St steel	3.03		
C-30 concrete		227.01	
C-35 concrete		263.96	
C-40 concrete		298.57	
C-45 concrete		330.25	
C-50 concrete		358.97	
Lightening		604.42	
Slab formwork			2.24

**Table 3:** Values of design variables obtained within the specified ranges.

Deck	Depth of the Deck (m)	Base Width (m)	Concrete Grade (MPa)	Deck	Depth of the Deck (m)	Base Width (m)	Concrete Grade (MPa)
1	1.65	3.65	35	16	1.55	4.10	35
2	1.70	3.80	45	17	1.25	3.50	45
3	1.20	3.85	40	18	1.40	3.30	40
4	1.55	3.60	45	19	1.45	3.90	45
5	1.20	4.85	50	20	1.35	3.60	35
6	1.15	4.50	50	21	1.50	3.35	45
7	1.35	3.95	30	22	1.50	4.50	45
8	1.30	4.45	30	23	1.55	3.20	30
9	1.35	4.25	45	24	1.25	3.00	50
10	1.50	4.55	30	25	1.40	3.45	45
11	1.60	4.20	40	26	1.50	3.55	35
12	1.25	4.70	40	27	1.70	3.85	45
13	1.50	4.05	45	28	1.20	3.60	40
14	1.45	4.35	35	29	1.30	4.90	40
15	1.65	3.45	45	30	1.45	4.75	35

**Table 4:** Energy cost of each of the analyzed decks.

Deck	Energy Cost (MW·h)	Deck	Energy Cost (MW·h)	Deck	Energy Cost (MW·h)
1	1149.88	11	1267.85	21	1134.93
2	1182.89	12	1191.65	22	1189.53
3	1065.87	13	1183.17	23	1103.41
4	1140.79	14	1119.17	24	1101.04
5	1170.72	15	1145.07	25	1201.73
6	1199.59	16	1162.92	26	1105.44
7	1103.18	17	1073.75	27	1165.47
8	1180.31	18	1152.33	28	1083.41
9	1132.71	19	1145.21	29	1215.82
10	1138.00	20	1094.86	30	1163.59

**Table 5:** Result after optimizing the search diversification phase.

Depth of the Deck (m)	Base Width (m)	Concrete Grade (MPa)	Energy Cost (MW·h)
1.15	3.35	40	1051.00

**Table 6:** Solutions and energy costs for each bridge deck in the intensification phase.

Deck	Depth of the Deck (m)	Base Width (m)	Concrete Grade (MPa)	Energy Cost (MW·h)
31	1.20	3.40	40	1059.87
32	1.15	3.90	35	1129.22
33	1.05	3.50	35	1237.89
34	1.10	3.80	45	1178.72
35	1.15	3.35	45	1074.77
36	1.25	3.60	45	1078.71
37	1.10	3.45	40	1124.21
38	1.20	3.35	45	1065.44
39	1.25	3.40	45	1084.92
40	1.15	3.60	45	1104.77

**Table 7:** Result post-optimization in the search intensification phase.

Depth of the Deck (m)	Base Width (m)	Concrete Grade (MPa)	Energy Cost (MW·h)
1.15	3.70	40	1038.28

## Data Availability

Not applicable.
